# Enhancing health research teams with CONTEXTperts

**DOI:** 10.1017/cts.2025.10229

**Published:** 2026-01-02

**Authors:** William R. Phillips

**Affiliations:** Department of Family Medicine, University of Washington, Seattle, WA, USA

**Keywords:** Advisory committees, consultants, public participation, stakeholder engagement, team science, translational research

## Abstract

CONTEXTperts (Context and Topic Experts) bring real-world experience in the context of the study topic and setting to enhance research team expertise, capacity, and creativity. They complement but do not replace authentic patient and community engagement, professional consultants, or formal advisory boards. Individual CONTEXTpert consultations or group meetings can help improve research questions, study designs, implementation plans, dissemination, and application of findings. They have added value to a variety of projects with varied research methods and study designs, including research with and without patient or community involvement. CONTEXTperts can bring vision, challenge, and reality checks to a variety of research teams with a practical, affordable model.

## Introduction

Research is increasingly done by teams that bring together complementary expertise, interests, and perspectives. Research teams often engage individuals beyond the core group to augment their knowledge, skills, and experience. Typically, they contract with research consultants who have specialist expertise in the study methods, specific clinical problems, populations, or interventions of the planned study. Research teams may also incorporate advisory groups to offer general expertise, guidance, and governance.

Research that aims to inform practice, policy, or public conversation is further empowered by engaging diverse voices of those who may be impacted by the research process or potential study findings. Research leaders in family medicine and primary care have pioneered advances in engaging the voices of patients, communities, and people with lived experiences that influence their care, health, and lives [1,2]. These models of engaged research usually apply to clinical or community health studies and require formal commitments and substantial resources.

Our experience with expanded research teams stimulated the addition of a different type of resource person – the CONTEXTpert. Contextperts are persons with understanding – knowledge, skills, and attitudes – earned through real-world experience with people, processes, and perspectives in the context where the research is conducted, or the study findings may be applied. They are not usually trained researchers. They need not be patients or community members and do not function as representatives or advocates. They are often people who work in the settings and with the people and problems that are the focus of the research but are not themselves the objects of the investigation or interventions.

## CONTEXTpert contributions

The aim of including contextperts is not to represent community voices but to enrich research questions, study processes, dissemination of findings, and applications of new knowledge. Integrating contextperts – as individuals or a small group – offers a limited, affordable, practical approach to expanding team capacity and creativity across a wide variety of studies. Contextperts can bring added value to all types of health research. They can enhance teams that already engage patients and community partners. They can also enrich teams whose research methods and study designs do not involve patients, communities, or populations.

Family medicine researchers are committed to engaging patients and communities in research that studies or affects them [1,2]. However, as generalist researchers, we also conduct a wide variety of studies that do not typically involve participatory or engagement models. The contextpert model can supplement all these research teams for planning, conducting, and reporting of a wide range of studies.

Contextperts roles differ from those of other key members of the research team. Table 1 outlines these complementary roles and contributions. Methodologists and content experts bring scientific, clinical, and research expertise and are often better placed as core research team members or consultants. Patients and community representatives bring lived experience and local knowledge of community relationships, priorities, and practicalities. In some models, their primary role is to represent the voices of those involved in the research process and affected by the research outcomes [1–4]. Advisory board members usually focus on governance, ethics, and equity [5].

**Table 1.** CONTEXTpert role compared to professional consultants, patients, and community representatives

Role	CONTEXTperts	Professional consultants	Patients community representatives
Representation	Minimal	Low	Primary role
Expertise	Observer insights local knowledge practicality problem solving	Methods content population clinical	Lived experience priorities acceptability practicality respect
Contributions	Contextual practical translation	Intellectual scientific internal validity	Practical justice external validity translation
Compensation	Honoraria variable volunteer	Employees subcontracts	Honoraria stipends subcontracts
Authorship	Uncommon	Usual	Variable

Compared to models of authentic engagement, the modest contextpert model requires commitment of fewer resources, less control, and simpler governance. It resembles a consultant model at the low end of the continuum of research engagement [6,7]. (For comparison to major models of research engagement, see Table S1, Supplement.) Contextperts can be helpful in more studies at less cost than in consultant, partnership, or advisory board models.

Contextpert voices complement but do not replace methodology and topic experts.

Adding contextperts to a research team does not replace the essential roles of patients, community members, and others in participatory research. Some studies can benefit from multiple models.

As part of our devotion to comprehensive care and generalist scholarship, we usually also include a patient, a practicing clinician, and a learner in all research teams.

## CONTEXTpert examples

Three examples can illustrate how research teams – even those thoughtfully assembled to include talented investigators, professional consultants, community advisory boards, and patient/participant partners – can still be enriched with the addition of contextperts with special local knowledge.

A community action research project to improve teen health through enhanced school lunch programs was designed by experienced researchers, with professional consultants in nutrition, dietetics, and teen mental health. It engaged community members and leaders, conducted teen focus groups, and sought advice from experts in the local culture. But it was a contextpert, a part-time cook at the off-site commercial kitchen that prepared school meals, that highlighted the practicalities of turning a scientifically designed monthly diet plan into daily meals prepared from available ingredients, cooked in the heat of the kitchen, delivered to the school, and served in the cafeteria line. It was another contextpert, the school custodian, who revealed which foods ended up in the garbage bin (∼40%), were thrown across the lunchroom (∼10%), or were traded for math homework. Contributions of these contexperts changed the program design and evaluation strategies.

A theoretical study in the prevention of sexually transmitted diseases by an interdisciplinary team of public health, sociology, and infectious disease specialists used state-of-the-art social network analysis to model patterns of sex partners and infection spread. No specific community members, patients, or study participants seemed to be needed for this computer modeling study. However, it was a contextpert family doctor who drew attention to how often, after divorce or separation, women have occasional (usually unprotected) sex with their ex-husbands, who themselves were having unprotected sex with multiple partners. It was a contextpert teenage boy living on the streets who unveiled the dynamics of a type of high-risk but usually unseen transactional sex work. The advice of these contextperts added dimensions to the social network model, expanding the diversity of risk behaviors considered and opportunities for prevention in the community.

A multi-method program of research on cancer prevention services in primary care, integrated interviews of practicing physicians and their patients, and medical record reviews to document practice patterns, beliefs, facilitators, and barriers to the use of flexible sigmoidoscopy in early cancer detection. The core research team included experts in behavioral theories of action, interviewing, focus groups, and surveys, and hired consultants in questionnaire design and distribution and statistical cluster analysis. It also engaged advisory groups of physicians and patients. But it was a contextpert medical office assistant who called out the important barriers created by physician office staff and their attitudes about cleaning the sigmoidoscopy equipment. This unexpected insight informed the recommendations for practice-based interventions to increase cancer detection.

Table 2 outlines a variety of other studies in which contextperts made substantial contributions to study planning, conduct, and outcomes.

**Table 2.** Examples of CONTEXTpert engagement in a variety of studies

Study type	Study topic	CONTEXTpert examples	Added insights
Epidemiologic study	Prevalence and patterns of suffering in family medicine [8]^a^	PC clinic receptionist PC medical assistant Hospital chaplain	Added PC patient problems involving suffering
Secondary data analysis	Training pathways to primary care in US medical school graduates [9]	Medical student career counselor Rural farmer patient DO physician	Changing DO training patterns and data sources
Practice-based research	Use of a new model of patient suffering in primary care [8]^a^	Spiritual counselor Medical Assistant Chronic illness researcher	Added language to elicit spiritual concerns
Randomized clinical trial	Effect of a reporting guideline on study publication [10]^a^	Research presenter Journal managing editor Research meeting staff	Added possible sources of bias in survey response

PC = primary care; DO = Doctor of Osteopathy.

^a^Study in progress.

In these examples and other studies, research teams found added value in the addition of contextperts with various knowledge, perspectives, and experiences. They brought crucial insights and information that meaningfully enhanced the design and results of the research and potentially empowered the implementation of study findings to achieve health goals.

## Working with CONTEXTperts

Based on experience across various research teams and studies, this outline offers practical guidance on designing, implementing, and working with contextperts and groups.

Simply asking the question, “Who else do we need around our team table?” can challenge and change the research question, team, and plan. Who does and understands this work? Who lives, works, and heals in this space? Who has deep knowledge, close to the ground, and near to the folks? Who has the map of the shortcuts and the landmines? Who cares? Who can help with the next steps in investigation, dissemination, or application?

### Recruiting and building groups

The professional identities, areas of expertise, and roles depend on the research question, design, and setting. Selecting contextperts is an opportunity to advance existing relationships and develop new ones. Contextpert diversity can cross many borders. Investigators can enlist other team members in the recruiting effort. One contextpert can fill several roles; developing a matrix may help coordinate selections.

Contextperts should ideally be included in the team early in the research process, ideally before finalizing the research question, study design, and plans for conducting the study. The earlier they are activated, the sooner they can help researchers shape vision, questions, plans, and choices. Individuals can be added later as needs and opportunities arise.

The research team can interact with the contextperts as individuals or as a group, depending upon the project needs. For most studies, working with contextperts as individuals is sufficient and efficient.

A group may offer advantages in efficiency and cross-fertilization. Building contextpert groups provides an opportunity to orchestrate complementary individuals, skill sets, and perspectives. Small groups of 5–7 members work well. Fewer restricts group dynamics and feels more like individual advisers or consultants. Larger groups create inefficiencies in communication. Contextperts need to be quickly responsive to help the team move forward.

Invitation and recruitment take time, making the early start important. It is an iterative process that requires adjusting each invitation to complement earlier recruits. Personal contacts from team members are more effective than group invitations. Each member deserves to feel valued. Early members may suggest potential members in a snowball process.

As with all research teams, it is important to be explicit about roles, time required, compensation, disclosures, and authorship. Clear communication aids recruitment and team process, with outlines of the research activities (Supplement Table S2) and member expectations (Supplement Table S3).

### Contextpert dynamics

The principal investigator or another research team member usually coordinates outreach to CONTEXTperts and facilitates any group meetings. Most interactions are via email or individual contacts. An early individual contact or meeting is valuable for orientation to the study, describing the contextpert role and group process, and encouraging ideas, questions, and challenges. A late contact or meeting before drafting the study report is valuable for discussing study findings, formulating final reports, planning dissemination, and discussing implementation. Contact may be intermittent throughout most of the study.

As with other research resource groups, contextperts and groups can be purpose-built for specific studies or developed to serve a broader research program. Over time, their interests and expertise may grow into more active roles on the research team. The contextpert model can be a strategy for building deeper and broader research capacity.

The model may not work for every study or research team. It can offer value to most health-related research, even for studies that do not involve systematic patient and community engagement. The value research teams get from contextperts is related to the value they place on listening, diversity, curiosity, and the desire to see their research improve health care, policy, and outcomes.

### Contextpert recognition

Service as described here does not meet the criteria for authorship [11], and these valuable resource people should understand that from the outset. (Occasionally, a member’s contributions may grow throughout the study to earn authorship.) Contextperts can be named in an acknowledgment section. Authors should consider publishing a special acknowledgment section that describes the group’s purpose, composition, contributions, and lists their names. This information might fit best in an appendix or online supplement to the published study report. (See an example acknowledgment in the Supplement.)

Contextperts deserve compensation for their expertise and time. Money might not be practical or required for some members. Consider including support for contextperts and any group activities in grant applications. Some funders may consider inclusion of contextperts an attractive asset for competitive research proposals.

Engagement of contextperts may be one way to meet the reporting standards of the CRISP Checklist for primary care research [12] and other EQUATOR research reporting guidelines [13].

## Discussion

We have found contextperts to be valuable additions to our research teams in a wide variety of studies: epidemiologic studies, secondary data analyses, practice-based research, randomized clinical trials, educational research, practitioner-led research, and learner research projects. (See examples, Table 2.)

The contextpert model is a modest extension of the commitment to engage more voices in more research. By applying the big concept of engagement to the modest model of contextperts, our teams have learned more from more people to improve more studies.

This model does not meet the commitments of formal engagement or participatory models [1, 2, 3]. However, this limited scope allows contextperts to be used widely across health research of more types with fewer concerns about resources, governance, ownership, and representation.

This model applies across many research methods and study designs. Some studies deserve patient partners; some require advisory panels. All studies can benefit from engaging broader viewpoints and varied voices.

The model is relatively cheap and easy; the major cost is the investment of curiosity, imagination, and humility. Recruiting, developing, and engaging contextperts or groups requires resources, but these are quite modest compared to the time, organization, and money needed for engagement with patient partners, community representatives, or formal advisory boards. Contextperts are cheaper than professional consultants but often bring more unique insights and perspectives.

Contextperts cannot replace authentic engagement or partnership with patients, communities, organizations, or the folks most involved as the subjects of the research or the objects of interventions. No outside experts can meet the need: “No research about us without us”[1].

These suggestions are based on limited experience with about ten studies and research teams. This experience does extend across a range of research methods, study designs, clinical problems, topics, and models of research engagement. Success has been assessed only by usefulness as judged by investigators and research team members. We have no objective measures of effectiveness for research outcomes.

In general, expanding research teams to engage a broader range of perspectives, experience, and expertise enriches the team and the research process. Contextperts can add new dimensions of diversity.

The strategic choice of contextperts is a practical challenge and an opportunity for creative thought. Contextperts contribute not representation, but reflection, vision, and perhaps a bit of disruption. We ask consultants and advisory groups to supply the expertise we know we need. We invite contextperts to offer insights we may not know we need. We can try to think outside the box. Contextperts help make the box bigger by pushing at the edges and corners.

Investing in and nurturing contextperts groups can bring new ideas to improve study planning, share the joy of curiosity, and empower research to improve care and health.

I recommend that researchers across the spectrum of health research experiment with adding contextperts to their research teams.

## Supporting information

10.1017/cts.2025.10229.sm001 Phillips supplementary material Phillips supplementary material

## Supplementary material

The supplementary material for this article can be found at https://doi.org/10.1017/cts.2025.10229.
