# Supplementary material for: Enhancing health research teams with CONTEXTperts
Source: J Clin Transl Sci. 2026 Jan 2;10(1):e13. doi: 10.1017/cts.2025.10229 (PMC12895443; doi:10.1017/cts.2025.10229)
Supplement: Phillips supplementary material [file S205986612510229Xsup001.docx]

**Enhancing Health Research Teams with CONTEXTperts**

William R. Phillips

**SUPPLEMENT**

**Table S1. Models of research engagement**

| **Engagement Model** | **Engagement**  **Continuum** [1] | **Resources**  **Time & Costs** | **Community**  **Power** | **Emphasis** | **Example**  **Proponents** |
| --- | --- | --- | --- | --- | --- |
| **CBPR**  Community-based participatory research [2] | **High**  Empower  .  .  .  Collaborate  .  .  .  Involve  .  .  .  Consult  .  .  .  Inform  **Low** | High  Capacity building  Infrastructure  Team training  Staff | High  Control of data and reports. | Relationship building.  Local benefit, equity, capacity building, sustainability.  All stages of research. | Primary care research NAPCRG [3] |
| **PAR**  Participatory Action Research [4] |  | High  (as above) | High  (as above) | Iterative cycles of action for local problem solving and change  (as above) |  |
| **PPI**  Patient and Public Involvement [5] |  | Medium  Training  honoraria | Variable | Explicit plans for patient and stakeholder engagement. | PCORI [6], AHRQ  NIHR [7] |
| **CEnR**  Community-Engaged Research [8] |  | Medium  Training  honoraria | Variable  Spectrum from consultation to co-leadership. | Governance embedded in institutional structures. | CTSA Cores [9]  IRBs [10] |
| **CAB**  Community Advisory Boards [11] |  | Low  honoraria  meetings | Low  Approval not ownership | Governance mechanisms  Ethical review | Hospitals, clinics, programs |
| **CONTEXTperts** |  | Lowest  Occasional  contact & meetings | Lowest  No authorship  No control of data or reports | Problem prediction and solving for conducting studies and applying findings. | Health research. Not limited to patient or community studies. |

Models overlap. Organizations may emphasize multiple or mixed models.

Abbreviations used in the table:

CTSA - Clinical and Translational Science Awards (USA)

IRB - Institutional Review Board (Human Subjects Protection)

NAPCRG – North American Primary Care Research Group

NIHR -National Institute for Health Research (UK)

PCORI - Patient-Centered Outcomes Research Institute (USA)

**Table S2. Recruitment outline for contextperts**

| Contextperts need transparent information on the following. |
| --- |
| 1. Outline of the research plan and study aims 2. Commitment of time (hours/month) and calendar schedule 3. Compensation offered (or clarity on no payment) 4. Who is on the research team? 5. Will there be a contextpert group, and who else will be serving on the group? 6. Involvement of others in the study: patients, clinicians, public 7. How will contextperts be recognized in reports and publications? 8. Study disclosures:    1. Ethical approval    2. Funding sources    3. Control of research, data, and publication decisions    4. Any restrictions: intellectual property, copyright |

**Table S3. Research team expectations of contextperts**

| 1. Read the research plan. 2. Review study materials. 3. Review drafts of study reports: abstracts, manuscripts, posters, and oral presentations. 4. Offer your feedback, questions, or suggestions. 5. Participate in two teleconferences, one at the start and one near the end of the project. 6. Read the final research reports. 7. Respond to occasional email questions. 8. Give written permission to list name in the acknowledgments section in research reports   List the estimated time required for each activity. |
| --- |

**Example of Published Acknowledgment for Contextperts**

To broaden the perspective of our study design and analysis, we engaged a diverse group of CONTEXTperts, individuals with local and topical experience, knowledge, and perspectives. They included seven individuals aged 23-60 years, five women, and four who described themselves as from underrepresented or marginalized backgrounds. Professional groups represented included: practicing family physician, academic family physician, farmer, MD physician, DO physician, health workforce policy maker, primary care policy fellow, medical student career counselor, public health trainee, patient representative, and educator. From the outset of the study, through data analysis and final report writing, the authors engaged these resource persons through meetings, email messages, surveys, updates, and draft revisions. The iterative process encouraged comments, feedback, pushback, new ideas, open criticisms, and fresh perspectives. The authors thank these colleagues for their valuable contributions: (option to list names). [Adapted from reference 12.]

**Supplement References**

1. **Key KD, Furr-Holden D, Lewis EY, *et al.*** The continuum of community engagement in research: a roadmap for understanding and assessing progress. *Prog Community Health Partnersh*. 2019;**13**(4):427-434. doi: 10.1353/cpr.2019.0064

2. **Collins SE, Clifasefi SL, Stanton J, The Leap Advisory Board**, ***et al.*** Community-based participatory research (CBPR): Towards equitable involvement of community in psychology research. *Am Psychol*. 2018;**73**(7):884-898. doi: 10.1037/amp0000167

3. **Allen ML Salsberg J, Knot M, *et al.*** *Engaging with communities, engaging with patients: Amendment to the NAPCRG 1998 Policy Statement on ethical research with communities.* North American Primary Care Research Group. 2015. https://www.napcrg.org/media/1270/2014pr.pdf. Accessed November 2, 2025,

4. **Macaulay AC**. Participatory research: What is the history? Has the purpose changed? *Fam Pract.* 2017:**34**(3):256-258. doi: 10.1093/fampra/cmw117

5. **Domecq JP, Prutsky G, Elraiyah T, *et al***. Patient engagement in research: A systematic review. *BMC Health Serv Res*. 2014;**14**:89. doi: 10.1186/1472-6963-14-89

6. **Sheridan S, Schrandt S, Forsythe L, Hilliard TS, Paez KA; Advisory Panel on Patient Engagement (2013 inaugural panel)**. The PCORI Engagement Rubric: promising practices for partnering in research. *Ann Fam Med.* 2017;**15**(2):165-170. doi: 10.1370/afm.2042

7. **Russell J, Greenhalgh T, Taylor M.** *Patient and public involvement in NIHR research 2006–2019: policy intentions, progress and themes*. NIHR Oxford Biomedical Research Centre. 2019.

8. **Michener L, Cook J, Ahmed SM, Yonas MA, Coyne-Beasley T, Aguilar-Gaxiola S**. Aligning the goals of community-engaged research: why and how academic health centers can successfully engage with communities to improve health. *Acad Med*. 2012;**3**:285-91. doi: 10.1097/ACM.0b013e3182441680

9. **Kubicek, K. and Robles, M**. *Resource for Integrating Community Voices into a Research Study: Community Advisory Board Toolkit.* Southern California Clinical and Translational Science Institute grant UL1TR001855. 2016. https//sc-ctsi.org/uploads/resources/CommunityAdvisoryBoard_Toolkit.pdf. Accessed November 2, 2025

10. **Mikesell, Bromley LE, Khodyakov D.** Ethical Community-Engaged Research: a literature review. ***Am J Public Health.* 103**;12: e7-e14. <https://doi.org/10.2105/AJPH.2013.301605>

11. **Newman SD, Andrews JO, Magwood GS, Jenkins C, Cox MJ, Williamson DC.** Community advisory boards in community-based participatory research: a synthesis of best processes. *Prev Chronic Dis*. 2011;**8**(3):A70,1-12.

12. **Phillips WR, Park J, Topmiller M.** Pathways to primary care: charting trajectories from medical school graduation through specialty training. *Health Aff (Millwood).* 2025;**44**(5):580-588. doi: 10.1377/hlthaff.2024.00893
